# Composite Dissolving Microneedles for Coordinated Control of Antigen and Adjuvant Delivery Kinetics in Transcutaneous Vaccination

**DOI:** 10.1002/adfm.201201512

**Published:** 2012-08-23

**Authors:** Peter C DeMuth, Wilfredo F Garcia-Beltran, Michelle Lim Ai-Ling, Paula T Hammond, Darrell J Irvine

**Affiliations:** Department of Biological Engineering, Massachusetts Institute of Technology77 Massachusetts Ave., Cambridge, MA 02139, USA; Program in Health Sciences and Technology, Massachusetts Institute of Technology77 Massachusetts Ave., Cambridge, MA 02139, USA; Department of Materials, Oxford UniversityOxford, UK; Department of Chemical Engineering, Koch Institute for Integrative Cancer Research, Institute for Soldier Nanotechnologies, Massachusetts Institute of Technology77 Massachusetts Ave., Cambridge, MA 02139, USA; Department of Materials Science and Engineering, Department of Biological Engineering, Koch Institute for Integrative Cancer Research, Massachusetts Institute of Technology77 Massachusetts Ave., Cambridge, MA 02139, USA; Ragon Institute of MIT, MGH, and HarvardBoston, MA 02139, USA; Howard Hughes Medical Institute4000 Jones Bridge Rd., Chevy Chase, MD 20815, USA

## Abstract

Transcutaneous administration has the potential to improve therapeutics delivery, providing an approach that is safer and more convenient than traditional alternatives, while offering the opportunity for improved therapeutic efficacy through sustained/controlled drug release. To this end, a microneedle materials platform is demonstrated for rapid implantation of controlled-release polymer depots into the cutaneous tissue. Arrays of microneedles composed of drug-loaded poly(lactide-*co*-glycolide) (PLGA) microparticles or solid PLGA tips are prepared with a supporting and rapidly water-soluble poly(acrylic acid) (PAA) matrix. Upon application of microneedle patches to the skin of mice, the microneedles perforate the stratum corneum and epidermis. Penetration of the outer skin layers is followed by rapid dissolution of the PAA binder on contact with the interstitial fluid of the epidermis, implanting the microparticles or solid polymer microneedles in the tissue, which are retained following patch removal. These polymer depots remain in the skin for weeks following application and sustain the release of encapsulated cargos for systemic delivery. To show the utility of this approach the ability of these composite microneedle arrays to deliver a subunit vaccine formulation is demonstrated. In comparison to traditional needle-based vaccination, microneedle delivery gives improved cellular immunity and equivalent generation of serum antibodies, suggesting the potential of this approach for vaccine delivery. However, the flexibility of this system should allow for improved therapeutic delivery in a variety of diverse contexts.

## 1. Introduction

Compared to more traditional routes such as oral administration and hypodermic injection, transcutaneous drug delivery through chemical permeation of the skin, iontophoresis, ultrasound, microneedle treatment, or various other strategies has the potential to provide many practical and clinical advantages.^1^ Relative to parenteral injection, transcutaneous delivery is non-invasive, potentially allowing for rapid, pain-free administration either by minimally trained health care providers, or through self-administration.^2^, ^3^ Transcutaneous delivery systems may reduce the generation of dangerous medical waste and inhibit the spread of disease known to occur through needle-reuse and needle-based injury.^4^, ^5^ Further, dry storage of systems designed for topical application may also provide enhanced drug stability, enabling transport of environmentally sensitive biological therapeutics without the need for refrigeration. This is a key issue as the requirement of “cold chain” distribution increases costs and inherently limits the availability of therapies throughout the developing world.^2^ Transcutaneous therapeutic administration also has the potential to enhance the clinical effectiveness of treatment, by allowing for more efficient delivery of drugs susceptible to first-pass metabolism in the liver.^3^

Recently, microneedle arrays have been employed as an enabling technology for safe and convenient transcutaneous delivery of diverse bioactive materials, including high molecular weight hydrophilic biologics, through pain-free mechanical disruption of the stratum corneum. For such barrier-disruption systems, rapid delivery of therapeutics into the skin by a brief application of the microneedle array is desirable, to minimize the potential risk of infection. This strategy has been demonstrated by various approaches, such as the use of microneedles that rapidly dissolve on entry into skin with release of encapsulated materials over minutes to hours.^6^–^16^ However, while rapid drug release allows a brief microneedle application time, bolus drug delivery by microneedles, such as bolus injections, can lead to rapid clearance of administered drugs and a need for large drug doses in order for released drug to remain at therapeutic doses (locally and/or systemically) for a sufficient temporal window to have a beneficial effect. Sustained or kinetically controlled drug release is more desirable than bolus delivery for many therapeutics and is essential to fulfill the full potential of transcutaneous delivery for sustaining drug levels within a therapeutic window over time. Previous strategies for obtaining controlled drug release with microneedle systems employed intradermal injection from hollow microneedles,^17^ or delivery from coatings on microneedle surfaces.^18^, ^19^ Alternatively, long term delivery has been achieved through encapsulation of drugs directly into biodegradable microneedles intended to remain in the skin for hours or days.^20^, ^21^ In each of these approaches, the microneedles must remain embedded in the skin, which raises the potential for infection in the setting of long-term/continuous drug delivery over days or weeks. To overcome this limitation, several strategies have been developed, including the use of microneedles to generate physical openings in the epidermis for topically applied controlled-release particle suspensions to diffuse into the skin,^22^ rapid delivery of drug-loaded polymer particles into the skin using dissolving microneedles,^23^ or fabrication of solid PLGA microneedles which rapidly fracture into implanted fragments upon skin insertion due to swelling of embedded hydrogel beads.^24^ The success of these approaches represents a strong proof of principle for the potential utility of microneedle platforms providing either rapid delivery or sustained release of therapeutics to the skin.

Vaccines have been shown to vary widely in potency based on the duration and kinetic profile of exposure to antigen and adjuvant combinations.^25^–^30^ Recently, we demonstrated that intra-lymph node injection of vaccines composed of soluble protein mixed with sustained-release adjuvant-loaded polymer particles could dramatically enhance the immune response to a model protein antigen.^30^ However, intranodal injection is a more complex procedure than traditional vaccine administration, and is best suited to vaccines against cancer or more specialized settings such as allergy treatment.^31^, ^32^ Here, we sought to determine whether control over antigen/adjuvant exposure kinetics could also enhance immune responses elicited by microneedle vaccines applicable to widespread prophylactic vaccination. We developed two parallel approaches for fabrication of composite dissolving microneedles combining a rapid release phase and sustained release phase for independently controlling the kinetics of antigen and adjuvant exposure during transcutaneous vaccination. These composite arrays were composed of poly(acrylic acid) (PAA) and poly (lactide-*co*-glycolide) (PLGA) in either a microparticle form or a bulk implant contained in the microneedle tip and intended for implantation within the skin upon application. PAA forms a glassy hard solid in bulk (Young’s modulus, *E* ≈ 4 GPa)^33^ but also dissolves rapidly in water. Exploiting this duality, we employed bulk solid PAA as a supportive matrix or pedestal as well as the base of the microneedle array itself. When inserted into skin, near-instantaneous dissolution of the PAA on hydration by interstitial fluid in skin provided a mechanism for rapid needle-tip disintegration or release and implantation in the cutaneous tissue. This approach enables delivery of diverse cargos that are hydrophilic or hydrophobic including small molecules or macromolecular drugs, either through encapsulation into PLGA particles or the PAA matrix. The selection of PLGA molecular weight or co-polymer ratio provides flexibility for tuning the kinetics of sustained cargo release and PAA encapsulation provides the additional ability for bolus delivery upon dissolution in vivo. These composite microneedle designs provide the ability for rapid administration leading to cutaneous implantation of controlled release depots for either bolus or sustained combinatorial release of therapeutics for various potential applications including vaccine delivery, as demonstrated here.

## 2. Results and Discussion

### 2.1. Fabrication of PLGA Microparticle - PAA Composite Microneedle Arrays

**Figure 1:**
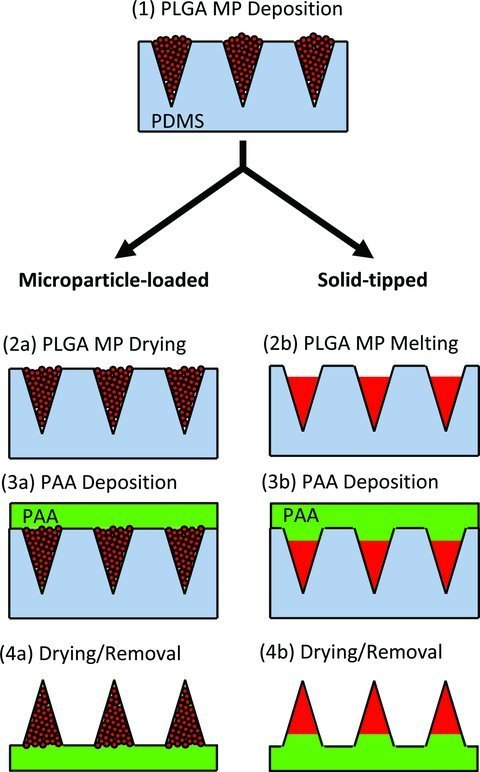
Schematic view of composite microparticle and bulk PLGA tip microneedle array fabrication. PDMS molds were first filled with PLGA microparticles through centrifugation (1). PLGA microparticles were then either dried in mold cavities (2a) or fused at high temperature to create a solid tip (2b). Concentrated aq. PAA solution was then centrifuged onto the filled molds to create a supportive matrix (3a) or pedestal (3b) for rapid dissolution in vivo. After drying microneedles were removed from PDMS molds (4a, 4b).

To create microneedle arrays capable of rapid simultaneous delivery of both soluble and PLGA-encapsulated cargos, we developed two parallel microneedle approaches intended to deposit either 1) PLGA microparticles or 2) solid PLGA implants into the superficial layers of the skin upon the aqueous dissolution of a supportive PAA matrix or pedestal. We hypothesized that such a platform would provide rapid administration through brief microneedle application while enabling combined bolus and long-term dosing of cargos released from cutaneously implanted PAA and PLGA depots, respectively. To fabricate the polymer composite microneedle structures needed to achieve these design goals, we employed a molding technique using polydimethylsiloxane (PDMS) molds. PDMS blocks were patterned with microscale cavities across their surface by laser micromachining, yielding a negative mold. In this strategy, cavity shape and the resulting microneedle geometry are easily controllable through variation of the laser micro-ablation process.^19^ We selected molds containing conical cavities, approximately 700 μm in height and 250 μm in width at the base. Microneedles of this size and shape have been shown to effectively penetrate the murine stratum corneum for cutaneous materials delivery^19^ and are also appropriate for delivery in humans. To begin the microneedle fabrication process, PLGA microparticles (1580 ± 95 nm in diameter, –26 ± 2 mV zeta potential) formed through double-emulsion-solvent-evaporation were applied to the surface of the mold in aqueous suspension and compacted into the mold cavities through centrifugation (**Figure 1**, step 1). Excess microparticles were then removed from the PDMS surface and the microparticle-loaded mold was allowed to dry (Figure 1, step 2a). To form dissolving microneedles carrying dispersible PLGA microparticles, we next added a concentrated aqueous solution of PAA (35 wt%) to the mold surface and infiltrated the PAA solution into the packed PLGA particle bed via centrifugation (Figure 1, step 3a). The loaded molds were then dried at 25 °C for 48 h before desiccation under vacuum to obtain solid PLGA-PAA microparticle matrices. The dry composite microneedles were finally removed from the PDMS mold for characterization or stored under vacuum at 25 °C until use (Figure 1, step 4a).

**Figure 2:**
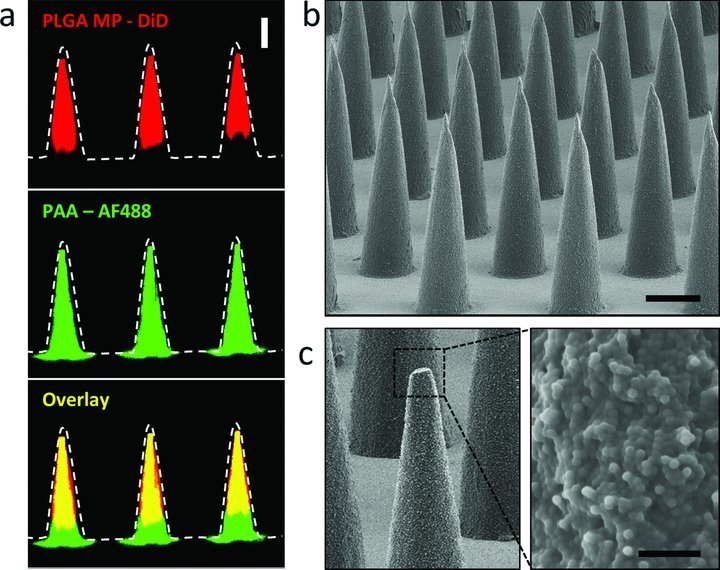
a) Confocal microscopy images of PLGA-PAA composite microneedles fabricated to encapsulate DiD-loaded PLGA microparticles (MP) (right, scale bar 200 μm). SEM images of b) resulting microparticle-encapsulating microneedle array (scale bar 200 μm) and c) high magnification image of the composite needle interior of a fractured microneedle (scale bar 10 μm).

To characterize the internal structure of microneedles created in this way, we performed the described fabrication steps using PLGA microparticles loaded with distinct lipophilic fluorescent tracers to serve as a model drug cargos: either 1,1′-dilinoleyl-3,3,3′,3′-tetramethylindocarbocyanine (DiI) or 1,1-dinoleyl-3,3,3′,3′-tetramethylindoc dicarbocyanine (DiD). Localization of the PAA matrix within the microneedle array was tracked through encapsulation of Alexafluor-488 (AF488) as a hydrophilic fluorescent tracer in the PAA matrix. The AF488 dye also served as a model for potential hydrophilic therapeutic compounds that could be encapsulated for bolus cutaneous delivery upon dissolution of the PAA matrix in vivo. Confocal imaging of microneedles fabricated using DiD-loaded PLGA microparticles together with AF488-loaded PAA showed uniform microneedle formation with DiD fluorescence localized in the microneedle tip, and AF488 fluorescence throughout the needle length (**Figure 2**a). This indicates the successful deposition of PLGA microparticles to the microneedle tip, and the effective formation of an encapsulating, supportive PAA matrix surrounding the microparticles and forming the base of the microneedle array itself. Further, confocal analysis demonstrated the flexibility of this approach through the similar successful fabrication of microneedles bearing multiple microparticle populations encapsulating either DiD or DiI in two different microparticle populations comprised of PLGA with high vs. low molecular weight (to obtain two distinct kinetics of drug release; see Figure S1a, Supporting Information). SEM imaging demonstrated the fidelity of the final composite microneedles to the mold cavity architecture (Figure 2b). Additional imaging of the internal structure of needles intentionally broken after fabrication also confirmed the confocal imaging results, showing the presence of nodules of discrete PLGA microparticles surrounded by PAA matrix (Figure 2c).

### 2.2. Fabrication of Solid PLGA-PAA Composite Microneedle Arrays

**Figure 3:**
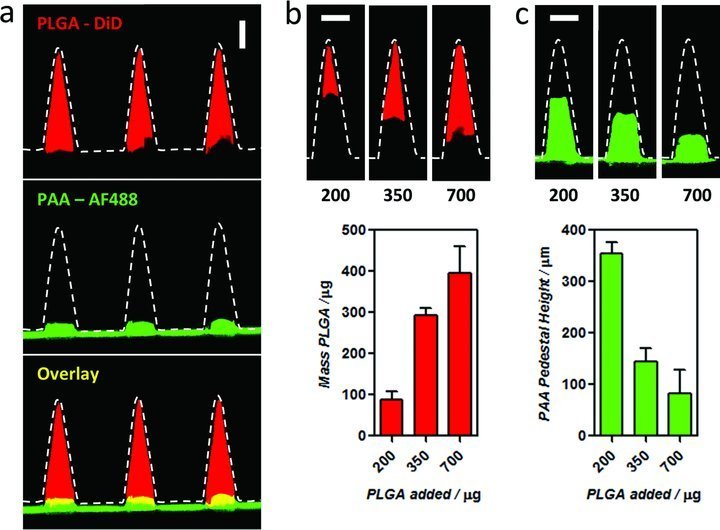
a) Confocal microscopy images of PLGA-PAA composite microneedles fabricated with a PAA base and DiD-loaded solid PLGA tip (scale bar 200 μm). Confocal microscopy images of microneedles fabricated with varying amounts of DiD-loaded PLGA microparticles as indicated giving a range of PLGA tip sizes (b) and PAA pedestal heights (c).

A similar fabrication approach was developed for the generation of microneedles capable of implanting solid PLGA tips directly into skin. As previously described, PLGA microparticles were first deposited into the cavities of a PDMS mold (Figure 1, step 1). After drying, the microparticle-loaded PDMS molds were incubated at 140 °C for 40 min under vacuum to fuse the embedded PLGA particles. The molds were then allowed to cool, solidifying the PLGA into solid cones at the tips of each mold cavity (Figure 1, step 2b). As before, concentrated PAA was then added and infiltrated into the PDMS molds through centrifugation, before drying and finally removal of the completed arrays (Figure 1, steps 3b and 4b). Confocal imaging of DiD-loaded PLGA together with AF488-loaded PAA showed the internal composite structure of the resulting microneedle arrays. In this case fluorescent signal from encapsulated DiD-loaded PLGA was localized to the tip of the microneedles, while AF488-PAA was only seen forming the base of the microneedle array (**Figure 3**a), consistent with the melted microparticles forming a continuous PLGA matrix in the microneedle tip, supported by a PAA pedestal at the microneedle base. Fabrication in which two distinct PLGA microparticle populations were sequentially added and melted in sequence yielded a layered microneedle structure in which the DiD-loaded PLGA added first formed the needle tip, while the remainder of the needle was composed of the second DiI-loaded PLGA layer, and finally the AF488-loaded PAA pedestal (Figure S1b, Supporting Information). Fine control over the size of the PLGA “warheads” (and therefore of encapsulated drug cargos) was readily achieved through simple variation of the mass of PLGA microparticles added to the mold. Confocal imaging analysis of microneedles fabricated using varying amounts of PLGA microparticles showed the formation of PLGA tips of increasing height and correspondingly shorter PAA pedestals as the mass of added PLGA particles was increased (Figure 3b,c). Control over PLGA tip size and PAA pedestal height is potentially an important parameter not only for controlling cargo dosage, but also for efficiency of delivery upon insertion to the skin.

### 2.3. In Vitro Testing of Composite Microneedle Delivery

**Figure 4:**
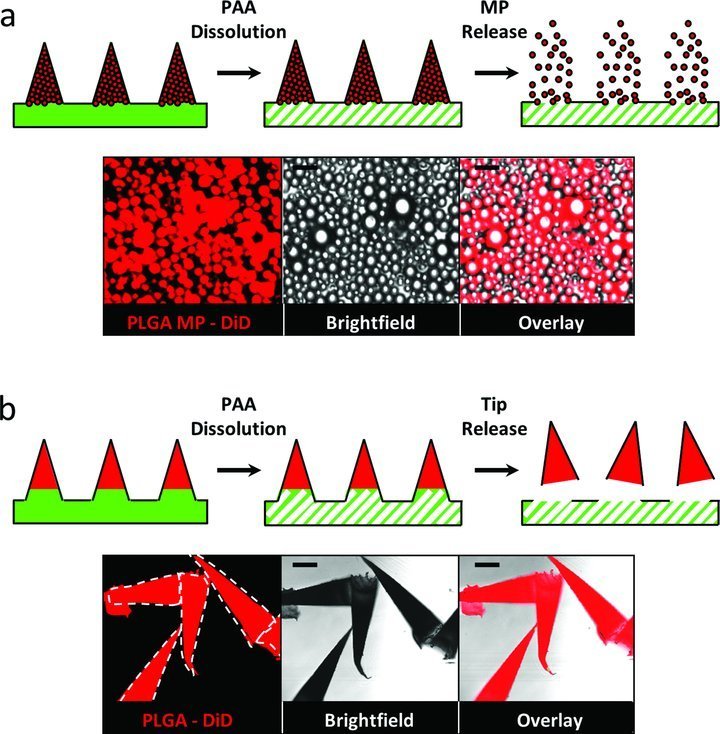
a) Schematic of PAA/microparticle composite microneedle dissolution and confocal microscopy image of DiD-loaded PLGA microparticles recovered following 5 s exposure of composite microneedles to PBS solution (scale bar 10 μm). b) Schematic of PLGA solid tip microneedle dissolution and confocal microscopy image of DiD-loaded PLGA microneedle tips recovered following 5 s exposure of solid tip microneedles to PBS (scale bar 200 μm).

To test our hypothesis that PLGA-PAA composite microneedles should be able to rapidly release either PLGA microparticles or solid PLGA tips upon exposure to interstitial fluids in the skin, we exposed composite microneedles to phosphate buffered saline (PBS) in vitro for short periods of time to observe the kinetics of PAA dissolution. When PAA microneedles encapsulating DiD-loaded PLGA particles were submerged in PBS, PAA dissolution was nearly instantaneous. After 5 s, the remaining microneedle array backing was recovered and the released microparticles were collected through centrifugation of the PBS solution for confocal imaging. The recovered particles were identical to those initially used for microneedle fabrication with no particle aggregation observed (**Figure 4**a). PAA microneedles encapsulating DiD-loaded bulk PLGA tip implants were similarly exposed to PBS and exhibited equally rapid separation from the residual PAA base after only 5 s. After centrifugation for collection, confocal imaging of the released PLGA tips showed retention of overall tip morphology (Figure 4b). Together these results suggested that the composite PLGA-PAA microneedle designs we had fabricated should be capable of rapidly depositing their PLGA payload upon dissolution of the supportive PAA matrix or pedestal in skin interstitial fluids.

### 2.4. In Vivo Composite Microneedle Delivery

**Figure 5:**
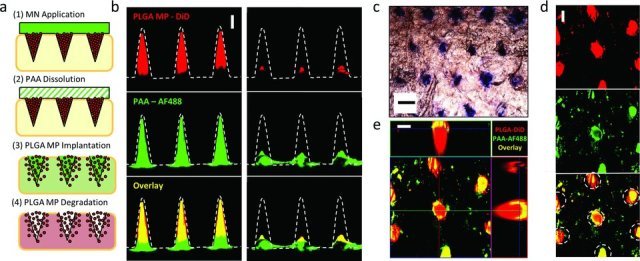
a) Microneedle delivery scheme: 1) Microneedle (MN) arrays are applied briefly to penetrate skin. 2) Cutaneous microneedle penetration exposes needles to interstitial fluid resulting in rapid dissolution of the supportive PAA matrix and microneedle disintegration. 3) Following microneedle base removal, microparticles are left behind at penetration sites where soluble PAA-encapsulated cargoes are rapidly delivered to the surrounding tissue. 4) Microparticle deposition into the skin establishes a depot for sustained delivery of PLGA encapsulated cargoes over time. b) Confocal microscopy image of DiD-microparticle-loaded microneedles before application (left) and following a 5 min application to murine skin (right, scale bar 200 μm). c) Optical microscopy image of microneedle-treated skin showing penetration sites stained using trypan blue (scale bar 500 μm). d) Confocal microscopy image of treated skin, showing deposition of DiD-loaded PLGA microparticles together with soluble AF488-loaded PAA at needle penetration sites directly following microneedle application for 5 min (scale bar 100 μm, penetration sites outlined). e) Reconstructed confocal *x*-*y*/*x*-*z*/*y*-*z* images depicting the microneedle application site showing deposition of microparticle-cargos within the cutaneous tissue (scale bar 100 μm).

Given the rapid in vitro release of both PLGA particles and solid PLGA tips from PLGA-PAA microneedles, we next sought to test the delivery capabilities of these composite designs in vivo. We hypothesized that composite microneedles encapsulating PLGA particles should have sufficient strength to penetrate the skin upon application (**Figure 5**a, step 1) before exposure to fluid in the cutaneous tissue would cause rapid dissolution of the PAA supportive matrix (Figure 5a, step 2). This would then lead to disintegration of the microneedle itself, releasing and implanting the encapsulated microparticles into the epidermis (Figure 5a, step 3). Then, after the residual microneedle backing was removed, implanted microparticles would remain as cutaneous depots for hydrolytic degradation and sustained release of their encapsulated cargos in situ (Figure 5a, step 4). We first tested AF488-PAA microneedles encapsulating DiD-loaded PLGA microparticles. To determine the efficiency and timescale of PAA dissolution and PLGA microparticle release, we applied microneedles to the dorsal ear skin of anesthetized C57Bl/6 mice. Following application to the skin, we performed confocal imaging on the post-application microneedle arrays to observe residual microneedle morphologies. Similar to the in vitro results, microneedle application to murine skin in vivo for 5 min was sufficient to produce nearly complete loss of both DiD-PLGA signal and AF488-PAA signal in the microneedle shaft, indicating rapid dissolution and disintegration of the composite microneedle matrix (Figure 5b). We stained the skin surface over the microneedle application site with trypan blue to visualize areas of stratum corneum disruption, and found uniform and consistent penetration of the composite microneedle arrays to the viable epidermis (Figure 5c). Confocal imaging analysis of dissected skin samples immediately following microneedle application indicated the presence of cutaneous microparticle depots at the sites of microneedle penetration, with colocalization of fluorescent signal from DiD-loaded PLGA microparticles and AF488 from the dissolved PAA matrix (Figure 5d). Using 3D rendering of confocal z-stacks, these depots were observed to extend 300–400 μm below the skin surface, consistent with the proposed paradigm for intact microneedle insertion, followed by PAA dissolution and microneedle disintegration, leaving depots of implanted material following removal of the microneedle backing (Figure 5e). Notably, we did not observe any short- or long-term local toxicity due to PAA exposure on the skin of treated animals, despite the relatively large chain length of the polymers used. This is likely due to the small amount of PAA (≤150 μg) introduced into the skin tissue through microneedle application, as well as the known low toxicity of PAA in commercial topical indications such as in detergents, cosmetics, or pharmaceutical use.^34^

We hypothesized a similar mechanism for delivery in the case of composite microneedles bearing bulk PLGA tips. As before, we theorized that intact composite needles would effectively insert into the outer layers of the skin before dissolution of the PAA pedestal by interstitial fluid, causing release and implantation of the PLGA tip for degradation and release of encapsulated cargos over time (Figure S2a, Supporting Information). Using AF488-PAA microneedles bearing DiD-loaded solid PLGA tips, we performed a similar set of tests to assay composite microneedle performance in vivo. As in the case of microparticle-loaded microneedles, application of composite microneedles bearing bulk PLGA tip implants for only 5 min to murine skin gave complete loss of DiD-PLGA signal as well as AF488-PAA signal from the patch backing, indicating PAA dissolution and needle tip separation (Figure S2b, Supporting Information). Trypan blue staining also confirmed that bulk PLGA tipped microneedles were able to penetrate skin consistently (Figure S2c, Supporting Information) following application for 5 min. Finally, confocal imaging of dissected skin following microneedle array application showed implantation of the PLGA microneedle tips following removal of the residual microneedle array base (Figure S2d, Supporting Information). As in the case of microparticle delivery, these bulk PLGA implants were observed 300–400 μm below the skin surface at the sites of microneedle penetration, together with fluorescent signal from the dissolved PAA pedestal (Figure S2e, Supporting Information).

### 2.5. Formation and Retention of Cutaneous Particle Depots for Sustained Cargo Release

**Figure 6:**
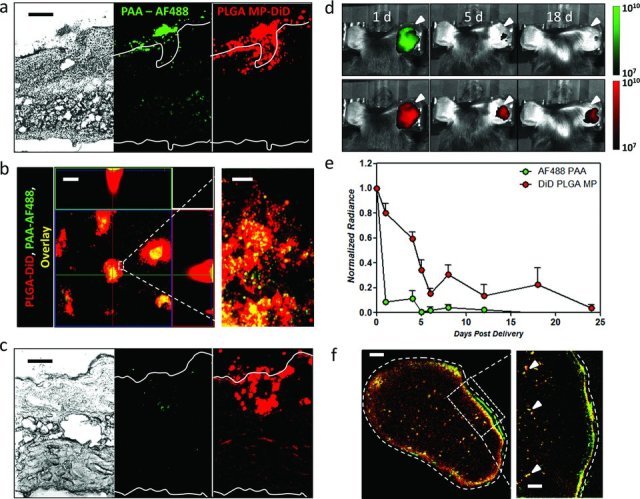
Composite DiD-loaded PLGA microparticle/AF488-loaded PAA microneedles were applied to the flank or dorsal ear skin of C57Bl/6 mice for 5 min, followed by histological, confocal microscope, and whole-animal fluorescence analysis at 1–18 days post treatment. a) Histological section of treated skin 24 h following microneedle array application. Microparticles are shown implanted together with AF488-loaded PAA at a single microneedle penetration site (scale bar 200 μm). b) Parallel *x*-*y*/*x*-*z*/*y*-*z* confocal reconstruction of the treatment site at 24 h post application shows DiD-loaded microparticle depots persisting at the penetration sites (left, scale bar 100 μm). High magnification imaging at a single penetration site shows microparticle dispersion within the cutaneous tissue (right, scale bar 20 μm). c) Histological section of skin 10 days following microneedle application (scale bar 200 μm). d) Whole animal fluorescence imaging of mice 1, 5, and 18 days after microneedle array application. Fluorescence signal from released AF488 and DiD-PLGA microparticles is shown. e) Quantification of relative AF488 and DiD whole-animal fluorescence signal detected at microneedle application sites. f) Histological section of the draining inguinal lymph node 10 days after microneedle application showing persistence of DiD PLGA-loaded cargos in the subcapsular sinus (left, scale bar 100 μm), as well as cell-trafficked PLGA particles in the cortical regions (arrows in inset at right, scale bar 10 μm).

We next sought to determine the fate of soluble PAA-loaded cargo and microparticle-loaded model drugs following deposition into the skin by composite microneedle application. As previously described, microneedles encapsulating DiD-loaded PLGA microparticles in an AF488-loaded PAA microneedle matrix were applied to the flank skin of C57Bl/6 mice for 5 min. Following treatment, individual animals were euthanized at various time points, and after dissection of the treatment site, confocal imaging and histological sectioning were performed to detect the presence of microparticle depots in the skin. Histological sectioning of tissue collected 24 h after treatment showed distinct cutaneous depots of microparticles extending several hundred micrometers below the skin surface, colocalizing with signal from PAA-encapsulated AF488 (**Figure 6**a). Similarly, confocal imaging of treated skin 24 h after administration indicated the retention of cutaneous microparticle deposits at the sites of microneedle insertion (Figure 6b). Consistent with the histological evidence, optical sectioning analysis of confocal data indicated the colocalization of DiD-labeled particle depots extending 300-400 μm beneath the skin surface. High magnification imaging of the depot site showed individual particles dispersed within the cutaneous tissue (Figure 6b). To determine whether the microneedle-implanted particles persist in the skin, we performed similar analyses of treated skin 10 days after microneedle administration. DiD-loaded microparticles were still found several hundred micrometers deep within the skin at this timepoint by histology (Figure 6c). However, tissue sections collected after 10 days consistently showed the loss of AF488 signal within the tissue at the sites of microneedle insertion, indicating the clearance of soluble PAA-loaded cargo over this time period.

Given the ability of composite microneedles to implant cutaneous microparticle deposits that were retained at the treatment site for long periods of time, we hypothesized that these microneedles might serve as an effective platform for sustained delivery of PLGA-loaded drugs, while also providing an opportunity to rapidly deliver bolus doses of additional cargos encapsulated in the PAA matrix. To determine the time course for cargo delivery following microneedle application, we performed composite microneedle treatments on the dorsal ear skin of C57Bl/6 mice as before, to deliver DiD-loaded PLGA microparticles together with PAA-loaded AF488. Tissue retention and clearance of these fluorescent model drug cargos was then monitored using whole animal fluorescent imaging. Results of this analysis showed that PAA-loaded AF488 was rapidly cleared from the site of application within 1 day, while DiD-fluorescent signal was retained within the ear skin for greater than 20 days following delivery (Figure 6d,e). These results are consistent with the previous confocal and histological analyses already discussed, and together support the hypothesized mechanism of microneedle insertion, followed by PAA dissolution, and needle disintegration to form long-lived cutaneous PLGA particle depots for sustained release over time. Similar whole animal imaging results obtained following delivery of bulk PLGA implants suggest an equivalent mechanism for composite microneedles of this variation (Figure S3, Supporting Information).

Nanoparticles and microparticles deposited in skin are known to be phagocytosed over time and transported to draining lymph nodes by antigen presenting cells.^35^–^37^ This transport process could affect the biodistribution of particle-released drugs and is an important factor in particle-based vaccines. To assess transit of particles from microneedle insertion sites to draining lymph nodes over time, confocal imaging of histologically sectioned draining lymph nodes was performed to detect the presence of microneedle-delivered cargos 10 days after treatment. These analyses clearly showed the presence of both AF488 (our model water-soluble PAA-encapsulated drug) and microparticle-loaded DiD within the subcapsular sinus of sectioned lymph nodes, suggesting these materials were able to drain through the lymphatics following release at the cutaneous depot site (Figure 6f). Further, intact microparticles were observed by high magnification imaging within the cortical regions of the lymph nodes, indicating trafficking of particles by cells from the implantation site. No fluorescent signal was observed in lymph nodes from untreated animals (data not shown). Together these results support the idea that cutaneously delivered soluble materials as well as microparticles themselves are trafficked to draining lymph nodes.

### 2.6. Composite Microneedle Subunit Vaccine Delivery

**Figure 7:**
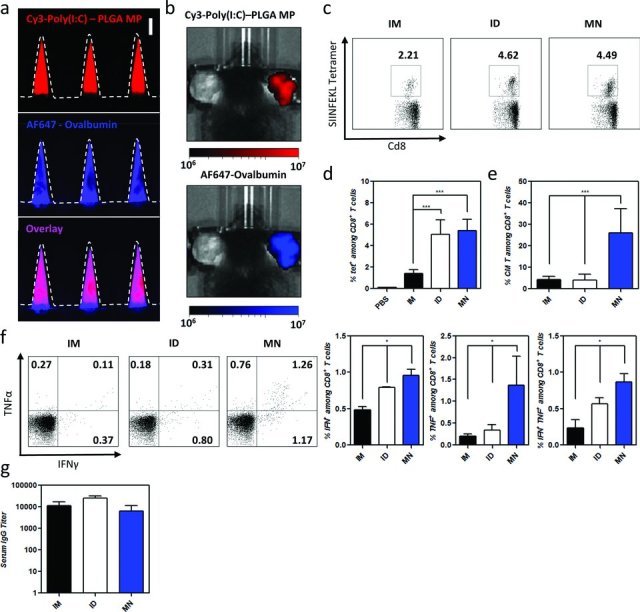
a,b) Composite microneedles were fabricated, comprised of Cy3-labeled poly(I:C)-loaded PLGA microparticles surrounded by PAA with entrapped AF647-ovalbumin. a) Confocal microscopy image of composite microneedles (right, scale bar 200 μm). b) Whole animal fluorescence imaging of mice treated with microneedles after 1 day. c–f) Groups of C57Bl/6 mice (*n* = 4) were vaccinated with 15 μg ovalbumin and 50 ng poly(I:C) intramuscularly (IM), intradermally in the dorsal ear skin (ID), or administered transcutaneously to the ear via microneedles (MN), with the poly(I:C) entrapped in PLGA microparticles and ovalbumin entrapped in the PAA phase. Mice were primed on day 0 and boosted on day 35 with identical formulations. c) Frequency of SIINFEKL-specific T cells in peripheral blood assessed by flow cytometry analysis of peptide-MHC tetramer^+^ CD8^+^ T cells. Shown are representative cytometry plots from individual mice and d) mean tetramer^+^ frequencies from day 49. e) Analysis of T-cell effector/central memory phenotypes in peripheral blood by CD44/CD62L staining on tetramer^+^ cells from peripheral blood. Shown are mean percentages of tetramer^+^CD44^+^CD62L^+^ among CD8^+^ T cells at day 63. f) Frequency of tetramer^+^ CD8^+^ T cells in peripheral blood producing IFN-γ and TNF-α following SIINFEKL restimulation assessed by flow cytometry. Shown are representative cytometry plots from individual mice and mean cytokine^+^ frequencies for day 56. g) Enzyme-linked-immunosorbent assay analysis of total ovalbumin-specific IgG in sera at day 63.

As a final test of the ability of composite microneedles to provide effective therapeutic delivery in vivo*,* we fabricated arrays for the transcutaneous delivery of a subunit vaccine formulation. We synthesized PLGA microparticles encapsulating Cy3-labeled poly(I:C) (Cy3-poly(I:C)), a synthetic double stranded RNA mimic of viral RNA, as a molecular adjuvant.^38^ Microneedles composed of poly(I:C)-loaded PLGA particles and fluorescently labeled ovalbumin (AF647-OVA, model antigen) embedded in a supporting PAA matrix were fabricated following the approach of Figure 1. Characterization of the resulting microneedles using confocal imaging showed restriction of the Cy3-poly(I:C) microparticles in the microneedle tips, with fluorescent signal from AF647-OVA throughout the microneedle length (**Figure 7**a). Upon application of these vaccine-loaded composite microneedles to the dorsal ear skin of mice, we observed successful deposition of both antigen and adjuvant at the application site using whole-animal fluorescence imaging (Figure 7b), consistent with our previous results using model drug cargos.

To test the efficacy of these dissolving composite microneedles for vaccination compared to traditional vaccine administration, mice were immunized on day 0 and boosted on day 35 with 15 μg ovalbumin and 50 ng PLGA-encapsulated poly(I:C) administered either intramuscularly (IM) in the quadriceps, intradermally (ID) in the dorsal ear skin, or by microneedle application (MN) for 5 min, also at the dorsal ear site. We then monitored the induction of OVA-specific cellular immunity through the detection of OVA-reactive CD8^+^ T cells using flow cytometry. Two weeks following the first immunization we observed expansion of antigen-specific CD8^+^ T cells to ≈2% of the total CD8^+^ population in all treatment groups, suggesting that microneedle delivery provided similar initial immunogenicity compared with traditional parenteral immunization strategies (Figure S4a, Supporting Information). Upon contraction of the antigen-specific T cell population two weeks later, we measured the frequency of antigen-specific CD44^+^CD62L^+^ cells, central memory T cells that have been correlated with long-lived effective protection against re-exposure to pathogens.^39^ On day 28, we observed that microneedle immunization generated central memory cells much more efficiently than animals receiving either intramuscular or intradermal vaccination, with greater than 30% of OVA-specific memory T-cells expressing CD44 and CD62L (Figure S4b, Supporting Information). These trends were similarly observed after the booster immunization, with antigen-specific T-cells reaching ≈5% of the total CD8^+^ population in mice receiving ID or MN dosing (Figure 7c,d). Similar to the pre-boost response, elevated frequencies of central memory CD8^+^ T cells were observed in microneedle vaccinated mice after contraction of the response on day 63, compared to both control IM and ID injected vaccine groups (Figure 7e). We also evaluated the functional capacity of the vaccine-elicited T-cell response through detection of cytokine production following re-stimulation of peripheral blood mononuclear cells with SIINFEKL peptide (the immunodominant peptide from OVA in C57Bl/6 mice). These analyses indicated enhanced cytokine production by T-cells elicited by microneedle vaccination for antigen-dependent production of both IFN-γ and TNF-α, key cytokines for combating viral infection (Figure 7f). Furthermore, microneedle delivery generated a higher frequency of multi-functional CD8^+^ T cells able to secrete both IFN-γ and TNF-α upon peptide stimulation. Together these results indicate the ability of microneedle immunization with slow-released adjuvant to elicit phenotypically and functionally superior cellular immunity compared to traditional vaccine administration. Finally, we measured the level of ovalbumin-specific IgG in the sera of immunized animals to compare the capacity for generating humoral immunity. Here we observed the presence of similarly high levels of anti-ovalbumin IgG in all immunized animals on day 63, suggesting that composite microneedle delivery can also generate antibody responses comparable to traditional immunization strategies (Figure 7g). Notably, the microneedles used in these vaccination studies were fabricated and stored dry at 25 °C for between 4 and 8 weeks before administration for both the prime and boost immunizations, indicating the potential for long-term maintenance of vaccine potency without the need for cold storage, a pressing need for the effective distribution of vaccines to remote areas of the developing world. Taken as a whole, these results demonstrate the strength of our approach for rapid transcutaneous multi-component delivery using composite PLGA-PAA microneedles.

## 3. Conclusions

Here we have demonstrated an approach for modular cargo encapsulation into composite dissolving microneedles for rapid delivery of PLGA microparticles or solid tips to form long-lived cutaneous depots promoting controlled and sustained release over several weeks. The combination of PLGA-formulated drug with a rapidly water-soluble PAA supporting matrix (in the case of microparticles) or pedestal (in the case of solid PLGA tips) provides the combined ability to rapidly and simply deliver the PLGA payload upon application to skin for only 5 min and to maintain drug release from implanted PLGA depots long after removal of the microneedle backing. Additionally, this composite microneedle architecture provides substantial flexibility in loading of single or multiple diverse cargos either into PLGA through standard double emulsion particle synthesis, or into the supporting PAA matrix through simple co-dissolution during fabrication. The composite structure also allows for simple tuning of extended cargo release kinetics through selection of PLGA copolymer ratio and molecular weight or bolus delivery upon needle disintegration through encapsulation in the PAA matrix. We have demonstrated the utility of these microneedle designs for the systemic dispersion of drugs released upon skin insertion or upon release from implanted PLGA over time. Finally, we have shown the successful application of this materials platform for encapsulation and delivery of a protein vaccine formulation generating potent humoral and cellular immunity matching or exceeding that resulting from traditional needle-based vaccine administration, even after storage for several weeks at room temperature. Together these results suggest that PLGA-PAA composite microneedles are an effective platform for straightforward and robust transcutaneous drug and vaccine delivery. Aside from applications in vaccination, the flexibility and modularity of this approach suggests its potential to serve as a platform for generalized therapeutics delivery providing both rapid and convenient administration as well as the potential for controlled and sustained systemic delivery.

## 4. Experimental Section

*PLGA Microparticle Synthesis:* Polyvinyl alcohol (PVA)-stabilized PLGA microparticles were prepared by a single/double emulsion/solvent evaporation approach. Briefly, 80 mg PLGA (50:50 lactide:glycolide ratio, IV 0.35 dL/g, Lakeshore Biomaterials or Resomer RG 755 S, IV 0.70 dL/g) was dissolved in 5 mL dichloromethane. The PLGA solution was then added to 40 ml 0.5% PVA (MW 150,000 Da, MP Biomedicals) while homogenizing at 12,000rpm using a T-25 Digital Homogenizer (IKA). Homogenization was performed for 3 min following PLGA addition, and solvent was subsequently removed by stirring overnight. For fluorescently labeled particles 160 ug 1,1′-dilinoleyl-3,3,3′,3′-tetramethylindocarbocyanine (DiI) or 1,1′-dioctadecyl-3,3,3′,3′-tetramethylindodicarbocyanine (DiD, Invitrogen) were codissolved with PLGA. For poly(I:C) loaded particles, 3mg Cy3-labeled poly(I:C) (Invivogen, labeled using Mirus LabelIT, according to the manufacturer’s instructions) was dissolved in distilled water and added to the polymer-containing organic phase while sonicating at 12W with a Microson XL probe tip (Microson). After sonication for 30 s the resulting emulsion was added to the PVA solution and homogenized as before. The resulting particles were collected by centrifugation, washed, and resuspended in distilled water before lyophilization and storage under dessication at 4 °C until use. Particle size and zeta potential was determined using a BIC 90+ light scattering instrument (Brookhaven Instruments Corp).

*Microneedle Fabrication:* Microneedles encapsulating free PLGA microparticles or bulk PLGA implants were fabricated from PDMS molds (Sylgard 184, Dow Corning) machined using laser ablation (Clark-MXR, CPA-2010 micromachining system) to create patterns of micron-scale surface-cavities. First, PLGA microparticles were deposited into PDMS molds through addition of aqueous particle suspensions to the mold surface. Following PLGA microparticle addition (0.2–3.0 mg/array), molds were centrifuged for 20 min at rcf ≈ 2 to compact particles into mold cavities. Following removal of residual material from the mold surface, molds were dried at 25 °C. For microneedles encapsulating free microparticles, addition of 35% poly(acrylic acid) (PAA, 250kDa) to the mold surface was followed by centrifugation (20 min, rcf ≈ 2) and drying at 25 °C (48 h on the benchtop, followed by 2–14 days under dessication), before removal. Microneedles encapsulating multiple distinct particle populations were fabricated similarly through addition of mixed particle suspensions. For microneedles encapsulating bulk PLGA implants, PDMS molds encapsulating free microparticles were heated under vacuum (–25 in. Hg) at 145 °C for 40 min, and then cooled at –20 °C before addition of 35% PAA, centrifugation, and drying as previously described. For microneedles encapsulating layered PLGA implants, sequential microparticle deposition, drying, and melting was performed as previously described. All microneedles were stored under dessication at 25 °C until use. Microneedle arrays were characterized by scanning electron microscopy (SEM) using a JEOL 6700F FEG-SEM and confocal microscopy using a Zeiss LSM 510.

*Characterization of Microparticle and Implant Delivery*: Microparticle and bulk implant release was characterized in vitro through brief (<30 s) exposure of fabricated arrays to PBS. Microparticles and bulk implants were then collected through centrifugation and washed in PBS before application of aqueous suspensions to glass coverslips. After drying, microparticles and implants were imaged by confocal microscopy. Similar delivery was measured in vivo following array application to the skin of mice. Animals were cared for in the USDA-inspected MIT Animal Facility under federal, state, local, and NIH guidelines for animal care. Microneedle application experiments were performed on anesthetized C57BL/6 mice (Jackson Laboratories) at the flank or dorsal ear skin. Skin was rinsed briefly with PBS and dried before application of microneedle arrays by gentle pressure. Following application mice were euthanized at subsequent time points and the application site and draining lymph nodes were dissected. Excised skin was stained with trypan blue before imaging for needle penetration. In separate experiments treated skin and applied microneedle arrays were imaged by confocal microscopy to assess transcutaneous delivery of encapsulated microparticles and bulk implants. In some cases, treated skin was excised and fixed in 3.7% formaldehyde for 18 h, then incubated in 30% sucrose/PBS for 2 h before embedding in optimal cutting temperature (OCT) medium (Tissue-Tek) for histological sectioning on a cryotome. Lymph nodes were similarly embedded in OCT and sectioned. Histological sections were then imaged using confocal microscopy.

*In Vivo Imaging*: Live whole animal imaging was performed using a Xenogen IVIS Spectrum (Caliper Life Sciences) on anesthetized mice. Fluorescence data was processed using region of interest (ROI) analysis with background subtraction and internal control ROI comparison to untreated skin using the Living Image 4.0 software package (Caliper).

*Immunizations*: All animal studies were approved by the MIT IUCAC and animals were cared for in the USDA-inspected MIT Animal Facility under federal, state, local, and NIH guidelines for animal care. Groups of 4 C57Bl/6 mice were immunized on days 0 and 35 with 15 μg ovalbumin and 50 ng poly(I:C) by intramuscular injection (15 μL in the quadriceps) intradermal injection (15 μL in the dorsal caudal ear skin) or by microneedle array (5 min application). Frequencies of OVA-specific CD8^+^ T-cells and their phenotypes elicited by immunization were determined by flow cytometry analysis of peripheral blood mononuclear cells at selected time points following staining with DAPI (to discriminate live/dead cells), anti-CD8α, anti-CD44, anti-CD62L (BD Biosciences), and phycoerythrin-conjugated SIINFEKL/H-2K^b^ peptide-MHC tetramers (Beckman Coulter). To assess the functionality of primed CD8^+^ T-cells peripheral blood mononuclear cells were stimulated ex vivo with 10 ug/mL OVA-peptide SIINFEKL for 6 h with Brefeldin-A (Invitrogen), fixed, permeabilized, stained with anti-IFNγ, anti-TNFα, and anti-CD8α (BD Biosciences), and analyzed by flow cytometry. Anti-Ovalbumin IgG titers, defined as the dilution of sera at which 450 nm OD reading was 0.25, were determined by ELISA analysis of sera from immunized mice. Animals were cared for following NIH, state, and local guidelines.

## Supporting Information

As a service to our authors and readers, this journal provides supporting information supplied by the authors. Such materials are peer reviewed and may be re-organized for online delivery, but are not copy-edited or typeset. Technical support issues arising from supporting information (other than missing files) should be addressed to the authors.
