# Supplementary material for: Composite Dissolving Microneedles for Coordinated Control of Antigen and Adjuvant Delivery Kinetics in Transcutaneous Vaccination
Source: Adv Funct Mater. 2012 Aug 23;23(2):161–72. doi: 10.1002/adfm.201201512 (PMC3595545; doi:10.1002/adfm.201201512)
Supplement: Supplementary file 1 [file adfm0023-0161-sd1.pdf]

Copyright WILEY-VCH Verlag GmbH & Co. KGaA, 69469 Weinheim, Germany, 2012.

ADVANCED FUNCTIONAL MATERIALS

Supporting Information

for *Adv. Funct. Mater.*, DOI: 10.1002/adfm. 201201512

Composite Dissolving Microneedles for Coordinated Control of Antigen and Adjuvant Delivery Kinetics in Transcutaneous Vaccination

*Peter C. DeMuth, Wilfredo F. Garcia-Beltran, Michelle Lim Ai-Ling, Paula T. Hammond, * and Darrell J. Irvine **

Submitted to ADVANCED FUNCTIONAL MATERIALS

DOI: 10.1002/adfm.201201512

Composite dissolving microneedles for coordinated control of antigen and adjuvant delivery kinetics in transcutaneous vaccination

By *Peter C. DeMuth, Wilfredo F. Garcia-Beltran, Michelle Lim Ai-Ling, Paula T. Hammond* and *Darrell J. Irvine*

[*] Corresponding authors Prof. D. J. Irvine and Prof. P.T. Hammond

P.C. DeMuth
Department of Biological Engineering
Massachusetts Institute of Technology
77 Massachusetts Ave, Cambridge, MA 02139 (USA)

W. F. Garcia-Beltran
Program in Health Sciences and Technology
Massachusetts Institute of Technology
77 Massachusetts Ave, Cambridge, MA 02139 (USA)

M. Lim Ai-Ling
Department of Materials
Oxford University
Oxford, United Kingdom

Prof. D.J. Irvine
Department of Materials Science and Engineering
Department of Biological Engineering
Koch Institute for Integrative Cancer Research
Massachusetts Institute of Technology
77 Massachusetts Ave, Cambridge, MA 02139 (USA)
Ragon Institute of MIT, MGH, and Harvard
Boston, MA 02139
Howard Hughes Medical Institute
4000 Jones Bridge Rd., Chevy Chase, MD
E-mail: djirvine@mit.edu

Prof. P.T. Hammond
Department of Chemical Engineering
Koch Institute for Integrative Cancer Research
Institute for Soldier Nanotechnologies
Massachusetts Institute of Technology
77 Massachusetts Ave, Cambridge, MA 02139 (USA)
E-mail: hammond@mit.edu

Keywords: (Drug Delivery, Nanoparticles, Biomedical Applications)

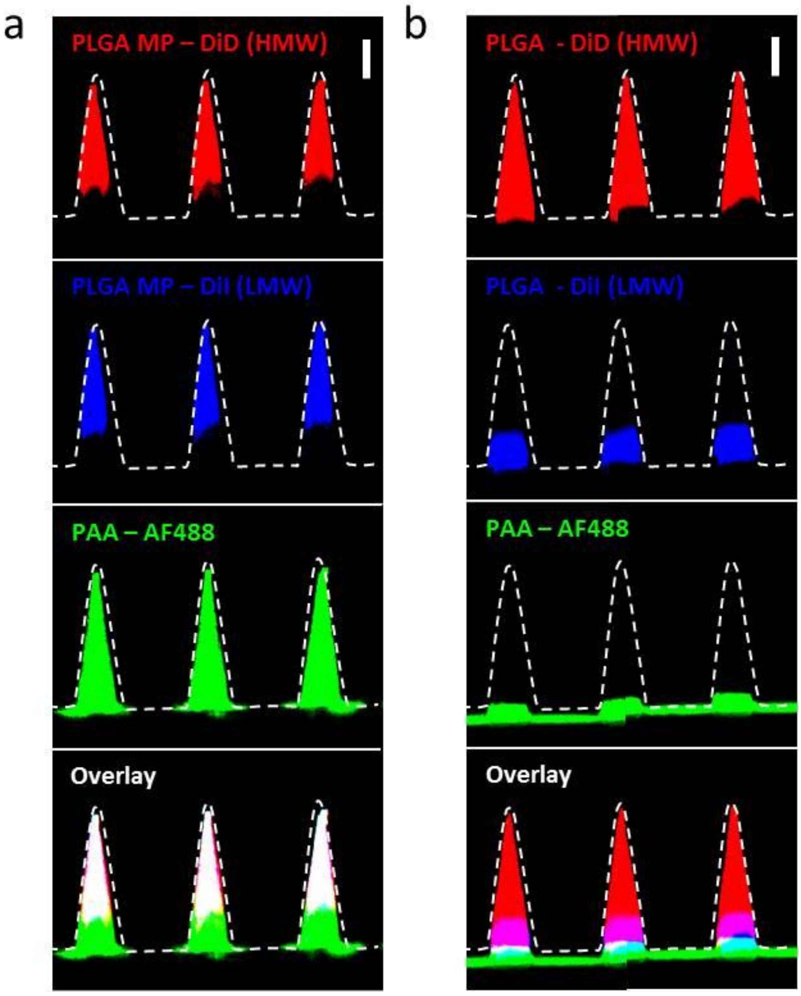

Figure S1. a) Confocal micrographs of PLGA-PAA composite microneedles fabricated to encapsulate both DiD- and DiI-loaded PLGA microparticles of high molecular weight (HWM) or low molecular weight (LWM) respectively (right, scale bar 200 µm) b) Confocal micrographs of resulting PLGA-PAA composite microneedles fabricated to encapsulate a layered DiI/DiD-loaded PLGA tip implant (scale bar 200 µm). DiD and DiI were encapsulated in PLGA microparticles synthesized from PLGA with IV 0.35 (LMW) or 0.70 (HMW).

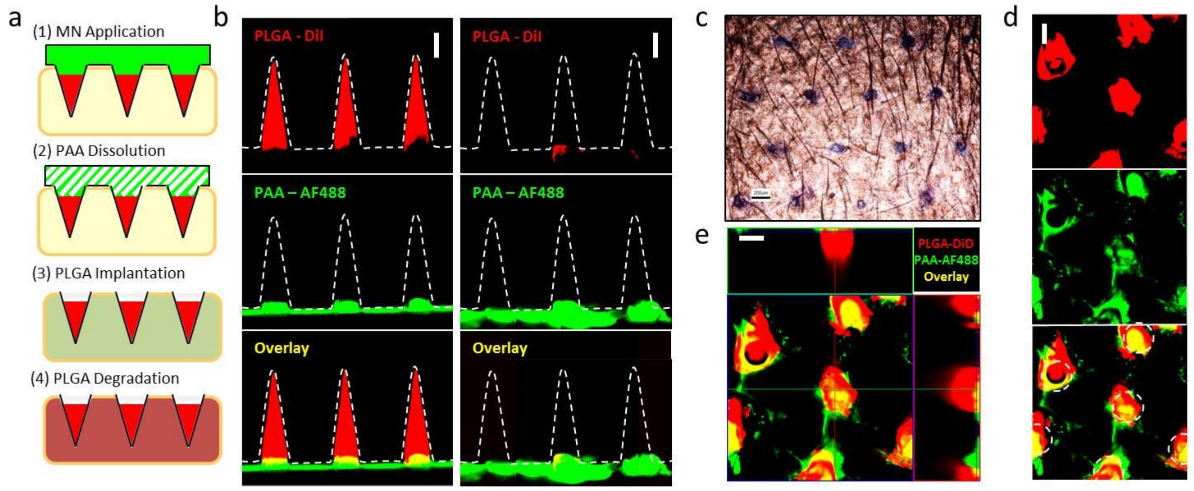

Figure S2. a) Microneedle delivery scheme: (1) Microneedle arrays are applied briefly to penetrate murine skin. (2) Cutaneous microneedle penetration exposes needles to interstitial fluid resulting in rapid dissolution of the supportive PAA pedestal. (3) Following microneedle base removal, released PLGA tips are left behind at penetration sites where soluble PAA-encapsulated cargoes are rapidly delivered to the surrounding tissue. (4) PLGA deposition into the skin establishes a depot for sustained delivery of encapsulated cargoes over time. b) Confocal micrographs of solid DiD-loaded PLGA tip microneedles before application (left) and following a 5 minute application to murine skin (right, scale bar 200 µm). c) Optical micrograph of microneedle treated skin showing penetration sites stained using trypan blue (scale bar 500 µm). d) Confocal micrograph of treated skin, showing deposition of DiD-loaded PLGA tip implants together with soluble AF488-loaded PAA at needle penetration sites directly following microneedle application for 5 minutes (right, scale bar 100 µm, penetration sites outlined). e) Reconstructed confocal z-stack depicting the microneedle application site showing deposition of PLGA-loaded cargoes within the cutaneous tissue (right, scale bar 100 µm).

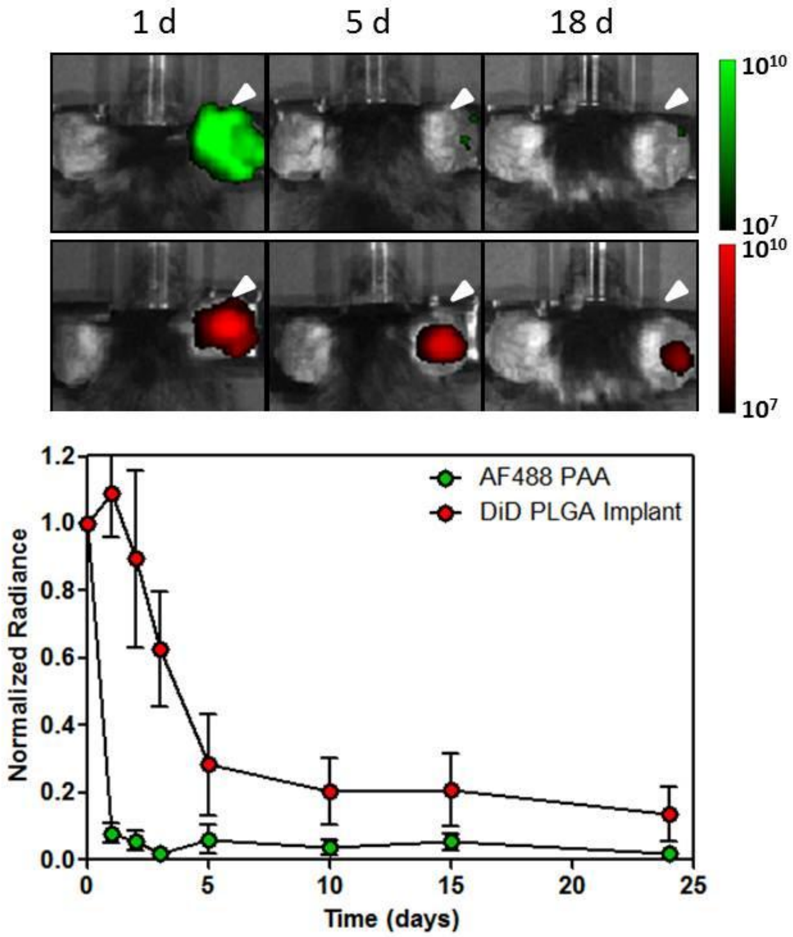

Figure S3. a) Whole animal fluorescence imaging of mice treated with DiD-loaded solid PLGA tip microneedles with AF488-loaded PAA pedestals after 1, 5, and 18 days. Fluorescence signal from AF488-PAA and DiD-PLGA implants is shown. b) Quantification of relative fluorescent signal detected at microneedle application site for bulk implant delivery.

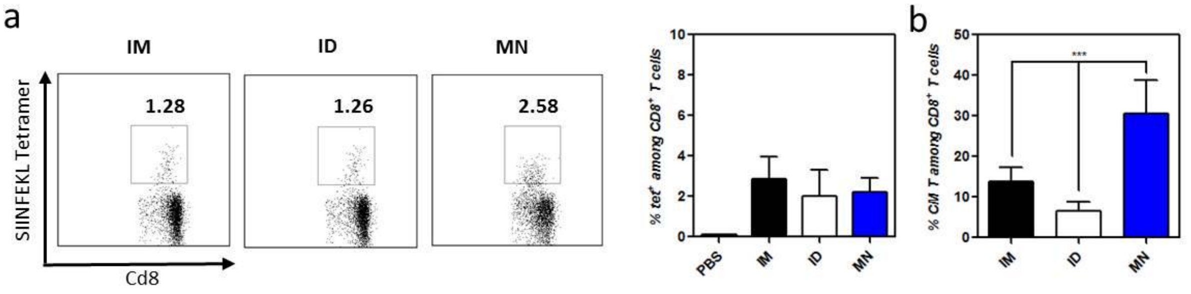

Figure S4. a) Frequency of SIINFEKL-specific T cells in peripheral blood assessed by flow cytometry analysis of tetramer$^+$ CD8$^+$ T cells. Shown are representative cytometry plots from individual mice and mean tetramer$^+$ frequencies from day 14. b) Analysis of T-cell effector/central memory phenotypes in peripheral blood by CD44/CD62L staining on tetramer+ cells from peripheral blood. Shown are mean percentages of tetramer$^+$CD44$^+$CD62L$^+$ among CD8$^+$ T cells at day 28.
